# Diversity and bioactive potential of culturable fungal endophytes of *Dysosma versipellis*; a rare medicinal plant endemic to China

**DOI:** 10.1038/s41598-018-24313-2

**Published:** 2018-04-12

**Authors:** Xiao-ming Tan, Ya-qin Zhou, Xiao-lei Zhou, Xiang-hua Xia, Ying Wei, Li-li He, Hong-zhen Tang, Li-ying Yu

**Affiliations:** 1 Guangxi University of Chinese Medicine, Nanning, 530200 China; 2 Guangxi Botanical Garden of Medicinal Plant, Nanning, 530023 China

## Abstract

The plant *Dysosma versipellis* is known for its antimicrobial and anticancer properties but is a rare and vulnerable perennial herb that is endemic to China. In this study, 224 isolates were isolated from various tissues of *D. versipellis*, and were classified into 53 different morphotypes according to culture characteristics and were identified by sequence analyses of the internal transcribed spacer (ITS) region of the rRNA gene. Although nine strains were not assignable at the phylum level, 44 belonged to at least 29 genera of 15 orders of Ascomycota (93%), Basidiomycota (6%), and Zygomycota (1%). Subsequent assays revealed antimicrobial activities of 19% of endophytic extracts against at least one pathogenic bacterium or fungus. Antimicrobial activity was also determined using the agar diffusion method and was most prominent in extracts from four isolates. Moreover, high performance liquid chromatography (HPLC) and ultra-performance liquid chromatography-quadrupole-time of flight mass spectrometry analyses (UPLC–QTOF MS) showed the presence of podophyllotoxin in two *Fusarium* strains, with the highest yield of 277 μg/g in *Fusarium* sp. (WB5121). Taken together, the present data suggest that various endophytic fungi of *D. versipellis* could be exploited as sources of novel natural antimicrobial or anticancer agents.

## Introduction

Resistance to antibiotics and drugs in pathogenic bacteria and fungi and overuse of antibiotics are the major challenges for researchers all over the world^1^. Thus, safer and novel antimicrobial drugs are eagerly awaited^2^, and natural secondary metabolites from endophytic fungi are increasingly considered due to their diverse structural classes and various bioactivities. These include antifungal^3^, antibacterial^4^, anticancer, anti-HIV^5^, and other promising bioactivities^6,7^. In addition, endophytic fungi are nontoxic and, thus, provide a promising source of novel drugs^8^.

Endophytic fungi inhabit living plant tissues without causing apparent disease or injury to the host^9^ and are ubiquitous in vascular plant species^10,11^. Currently, less than 10% of the approximately one million known terrestrial endophytes have been investigated^12^. However, several rare medicinal plants produce important bioactive compounds to survive in unique environments and may host novel and diverse fungal endophytes^7,13^, and these have rarely been isolated and characterized.

**Figure 1:**
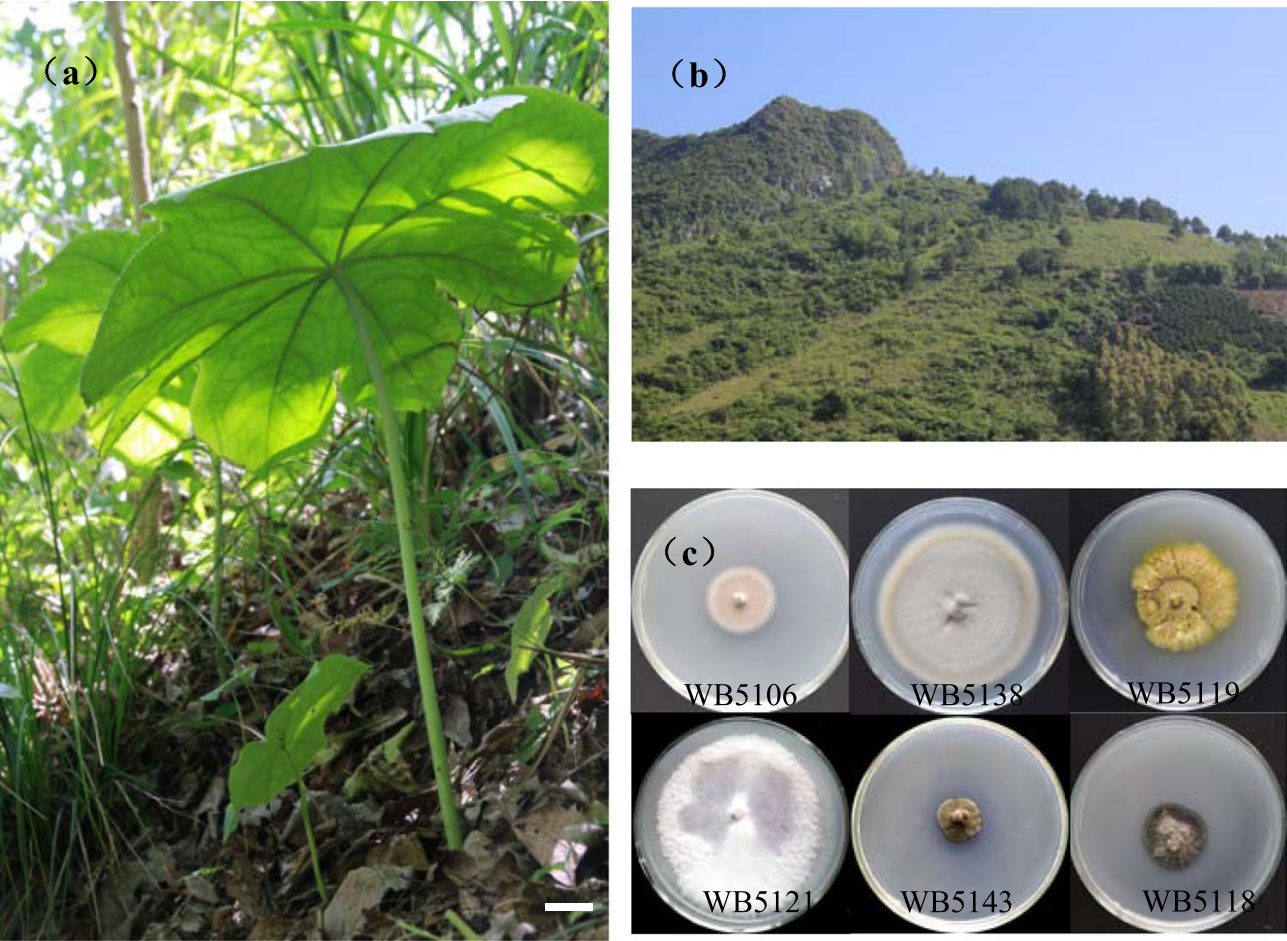
Habitat of *D. versipellis* and its endophytic fungi. Adult plants of *D. versipellis* (Bar = 20 mm; (**a**) growing among hillside shrubs (**b**) and representative fungal morphotypes isolated from *D. versipellis* growing on potato dextrose agar (PDA) for 2 weeks at 26 °C (**c**).

*Dysosma versipellis* (Hance) M. Cheng ex Ying (Fig. 1a) is commonly referred to as podophyllum, hemipilia, fatsia, or octagonal lotus, and is a rare and vulnerable perennial herb of the Berberidaceae family^14^. This plant species is endemic to China and is mainly distributed in high altitudes ranging from 200–2400 m above sea level in disjunct stands of warm-temperate, deciduous, montane forests (Fig. 1b) across central and eastern China^15^. *Dysosma* species including *D. aurantiocaulis*, *D. difformis*, *D. majorensis*, *D. pleiantha*, *D. tsayuensis*, *D. veitchii*, and *D. versipellis* have been identified in previous studies and six of these are endemic to China^16^. As a traditional Chinese medicine, extracts from the rhizomes of this plant has been used as antibacterial treatments for syphilis and an antidote for snake bites^17^. In recent decades, *D. versipellis* has attracted increasing pharmaceutical attention due to the discovery of podophyllotoxin (PTOX), which is a pivotal lignan and is used as a natural source of various anticancer PTOX derivatives^18^. Recent studies show antiviral and anti-inflammatory properties of the flavonoids quercitrin and kaempferol from this plant^19^. However, due to overexploitation and slow growth, all *Dysosma* species have been under the threat of extinction^20^. Therefore, to protect these valuable medicinal plants and maintain the supply of PTOX for anticancer drugs, alternative sources are eagerly sought. Among these, endophytic fungi have the potential to produce PTOX^21^ for the production of podophyllotoxin^21^. However, to date, only a few PTOX-producing fungi associated with Berberidaceae plants have been reported^22,23^. In the present study, we investigated the diversity of culturable fungal endophytes of *D. versipellis* and screened the endophytic fungi for antimicrobial activities and PTOX-producing fungal isolates using HPLC and UPLC–QTOF MS analyses.

## Results and Discussion

### Isolation, sequencing data, and diversity of culturable endophytic fungi

In this study, a total of 224 fungal colonies (isolation rate, 41.2%) were isolated from 544 tissue segments of *D. versipellis* plants and included 62 (32.6%), 104 (42.3%), 33 (73.3%), and 25 (39.7%) strains from root, rhizome, stem, and leaf tissue segments, respectively (Table 1). The 224 isolates were assigned to 53 representative morphotypes (19, 22, 6, and 6 strains from roots, rhizomes, stems, and leaves, respectively) according to culture characteristics on potato dextrose agar (PDA; Fig. 1c), and all culturable morphotypes were identified according to ITS rDNA sequence analyses. Subsequently, 44 isolates were categorized at the genus level based on sequence similarity analyses, and the other nine isolates remained unidentified due to low sequence homology in the GenBank database.

**Table 1:** Endophytic isolates from *D. versipellis* tissues.

Tissues	Segments examined	Segments infected	Total isolates	Endophytic species	Total CR%	Total IR%	Shannon_*H′*
Root	190	58	62	19	30.50%	32.60%	2.433
Rhizome	246	97	104	22	39.40%	42.30%	2.728
Stem	45	31	33	6	68.80%	73.30%	1.330
Leaf	63	23	25	6	36.50%	39.70%	1.242
Total	544	209	224	53			

According to diversity and sequence data of 53 isolates recovered from *D. versipellis* plants, at least 29 fungal genera were identified (Table 2). Among these, 25 belong to the Ascomycota and the isolates matched to 32 different species. The isolates WB5104 and WB5105 were identified only at the phylum level, and belonged to Ascomycota. Four (7.5%) isolates were classified as Basidiomycota, comprising the genera *Phyllosticta* (WB5139), *Psathyrella* (WB5140) and *Rhizoctonia* (WB5145 and WB5146). One (1.9%) isolate (WB5130) was classified as Zygomycota, and the genus *Mucor*. Shannon–Wiener diversity indices (*H′*; Table 1) show that *D. versipellis* host various fungal species, and that their rhizome tissues have the highest endophytic community diversity (2.728), followed by their roots (2.433), stems (1.330), and leaves (1.242).

**Table 2:** Culturable endophytic fungi from *D. versipellis* and corresponding isolation rates (IR%).

Fungal isolate	Accession number	Closest relatives in NCBI	ITS identity (%)	Tissue	IR %	Phylum; Class; Order	Classification
WB5101	KY940469	*Acremonium nepalense* CBS 113254 (DQ825972)^24^	99	Leaf	1.12	Ascomycota; Sordariomycetes; Glomerellales	*Acremonium* sp.
WB5102	KY940470	*Alternaria alternata* CBS 112018 (AY673074)^25^	99	Root	0.53	Ascomycota; Dothideomycetes; Pleosporales	*Alternaria* sp.
WB5103	KY940471	*Arthrinium arundinis* CBS 114316 (KF144884)^46^	99	Root	0.53	Ascomycota; Sordariomycetes; Xylariales	*Arthrinium* sp.
WB5104	KY940472	Ascomycota P7 (AY265338)^47^	80	Rhizome	0.41	Ascomycota	Ascomycota
WB5105	KY940473	Ascomycota (JX427054)^48^	84	Root	0.53	Ascomycota	Ascomycota
WB5106	KY940474	*Cladosporium uredinicola* SACCR 040661 (AY251071)^49^	99	Rhizome	0.41	Ascomycota; Dothideomycetes; Capnodiales	*Cladosporium* sp.
WB5107	KY940475	*Colletotrichum excelsum-altitudum* CGMCC 3.15131 (JX625182)^50^	99	Leaf	1.12	Ascomycota; Sordariomycetes; Glomerellales	*Colletotrichum* sp.
WB5108	KY940476	*Colletotrichum gigasporum* P1982 (KT269249)^51^	99	Stem	2.22		*Colletotrichum* sp.
WB5109	KY940477	*Colletotrichum gloeosporioides* CBS 119204 (JX010150)^52^	99	Leaf	19.04		*Colletotrichum* sp.
WB5110	KY940478	*Colletotrichum karstii* CGMCC 3.15123 (JX625163)^50^	99	Leaf	1.12		*Colletotrichum* sp.
WB5111	KY940479	*Colletotrichum siamense* GM29 (KC512127)^53^	100	Stem	2.22		*Colletotrichum* sp.
WB5113	KY940481	*Cylindrocarpon liriodendra* CBS 117640 (DQ178166)^54^	99	Rhizome	0.41	Ascomycota; Sordariomycetes; Hypocreales	*Cylindrocarpon* sp.
WB5114	KY940482	*Cylindrocarpon pauciseptatum* Cy196 (JF735305)^55^	99	Root	0.53		*Cylindrocarpon* sp.
WB5131	KY940498	*Cylindrocarpon* sp. YIMPH30026 (KP230827)^56^	97	Root	0.53		*Cylindrocarpon* sp.
WB5115	KY940483	*Dactylonectria alcacerensis* CBS 129087 (NR_121498)^55^	99	Rhizome	0.41	Ascomycota; Sordariomycetes; Hypocreales	*Dactylonectria* sp.
WB5149	KY940504	*Uncultured Diaporthales* R77p1 (GU327455)^57^	94	Root	0.53	Ascomycota; Sordariomycetes; Diaporthales	Diaporthales
WB5116	KY940484	*Diaporthe perjuncta* CBS 109745 (KC343172)^58^	96	Rhizome	0.41	Ascomycota; Sordariomycetes; Diaporthales	*Diaporthe* sp.
WB5117	KY940485	*Diaporthe* sp. HKB37 (DQ092525)^59^	96	Rhizome	0.41		*Diaporthe* sp.
WB5118	KY940486	*Exophiala* sp. AS29-1 (AB752282)^60^	99	Rhizome	3.65	Ascomycota; Eurotiomycetes; Chaetothyriales	*Exophiala* sp.
WB5120	KY940488	*Fusarium nematophilum* BBA 70838 (HQ897786)^45^	99	Rhizome	2.43	Ascomycota; Sordariomycetes; Hypocreales	*Fusarium* sp.
WB5121	KY940489	*Fusarium oxysporum* ERP-10 (JN222394)^61^	99	Root	0.53		*Fusarium* sp.
WB5122	KY940468	*Fusarium solani* ATCC 56480 (FJ345352)^62^	100	Root	0.53		*Fusarium* sp.
WB5123	KY940490	*Hypoxylon fragiforme* 22 (JN198512)^63^	99	Rhizome	0.41	Ascomycota; Sordariomycetes; Xylariales	*Hypoxylon* sp.
WB5124	KY940491	*Ilyonectria coprosmae* CBS 119606 (JF735260)^55^	96	Root	10.53	Ascomycota; Sordariomycetes; Hypocreales	*Ilyonectria* sp.
WB5125	KY940492	*Ilyonectria macrodidyma* K6 (JF807395)^64^	99	Rhizome	1.62		*Ilyonectria* sp.
WB5126	KY940493	*Ilyonectria robusta* CBS 117815 (JF735266)^55^	96	Rhizome	5.69		*Ilyonectria* sp.
WB5127	KY940494	*Ilyonectria torresensis* CBS 112598 (JF735351)^55^	99	Rhizome	1.62		*Ilyonectria* sp.
WB5128	KY940495	Leotiomycetes AK1466 (JQ759764)^65^	89	Root	0.53	Ascomycota; Leotiomycetes	Leotiomycetes
WB5129	KY940496	*Minimelanolocus aquaticus* 15–0414 (KR215607)^66^	97	Rhizome	2.03	Ascomycota; Eurotiomycetes; Chaetothyriales	*Minimelanolocus* sp.
WB5130	KY940497	*Mucor* sp. CY118 (HQ607969)^67^	95	Root	0.53	Zygomycota; Zygomycetes; Mucorales	*Mucor* sp.
WB5132	KY940499	*Ochroconis* cf. constricta CBS 124172 (GQ426969)^68^	99	Leaf	1.12	Ascomycota; Dothideomycetes; Venturiales	*Ochroconis* sp.
WB5133	KY940500	*Ophioceras* sp. F2224 (KU747946)^69^	94	Stem	2.22	Ascomycota; Sordariomycetes; Magnaporthales	Magnaporthales
WB5119	KY940487	OphiostomatalesF1732 (KU747803)^69^	97	Stem	2.22	Ascomycota; Sordariomycetes; Ophiostomatales	Ophiostomatales
WB5134	KY940501	*Microsphaeropsis* sp. S4A1ACS (KY305064)^70^	99	Root	0.53	Ascomycota; Dothideomycetes; Pleosporales	*Microsphaeropsis* sp.
WB5135	KY940502	*Pestalotiopsis oryzae* CBS 111522 (KM199294)^71^	99	Root	0.53	Ascomycota; Sordariomycetes; Xylariales	*Pestalotiopsis* sp.
WB5136	KY940503	*Phialophora mustea* BAN-C4 (JN123359)^72^	99	Root	0.53	Ascomycota; Eurotiomycetes; Chaetothyriales	*Phialophora* sp.
WB5137	KY940505	*Phoma putaminum* CBS 372.91 (GU237843)^73^	99	Root	0.53	Ascomycota; Dothideomycetes; Pleosporales	*Phoma* sp.
WB5138	KY940506	*Phoma selaginellicola* CBS 122.93 (GU237762)^73^	99	Root	0.53		*Phoma* sp.
WB5139	KY940507	*Phyllosticta* sp. MUCC0547 (AB454364)^74^	99	Rhizome	0.41	Basidiomycota; Agaricomycetes; Agaricales	*Phyllosticta* sp.
WB5140	KY940508	*Psathyrella candolleana* P73 (AM712281)^75^	99	Rhizome	0.41	Basidiomycota; Agaricomycetes; Agaricales	*Psathyrella* sp.
WB5141	KY940509	*Pseudocercospora humuli* CPC 11358 (GU214676)^76^	99	Stem	26.7	Ascomycota; Dothideomycetes; Capnodiales	*Pseudocercospora* sp.
WB5112	KY940480	*Pyrenochaeta* sp. P2916 (KT270113)^51^	98	Root	1.05	Ascomycota; Dothideomycetes; Pleosporales	*Pyrenochaeta* sp.
WB5142	KY940510	*Pyrenochaeta* sp. CBS 135108 (KF251149)^77^	97	Leaf	1.12	Ascomycota; Dothideomycetes; Pleosporales	*Pyrenochaeta* sp.
WB5143	KY940467	*Ramichloridium* sp. NC1_3.3F1a (FJ425199)^78^	96	Stem	2.22	Ascomycota; Dothideomycetes; Capnodiales	*Ramichloridium* sp.
WB5144	KY940511	*Rhexocercosporidium* sp. Dzf14 (EU543257)^79^	99	Rhizome	0.41	Ascomycota; Leotiomycetes; Helotiales	*Rhexocercosporidium* sp.
WB5145	KY940512	*Rhizoctonia* sp. Rh183 (JF519833)^80^	99	Rhizome	0.81	Basidiomycota; Agaricomycotina incertae sedis	*Rhizoctonia* sp.
WB5146	KY940513	*Rhizoctonia* sp. R14 (AY927321)^81^	95	Root	0.53		*Rhizoctonia* sp.
WB5147	KY940514	SordarialesREF169 (JN859389)^82^	95	Root	2.11	Ascomycota; Sordariomycetes; Sordariales	Sordariales
WB5148	KY940515	Sordariomycetes AK0924 (JQ759304)^65^	88	Rhizome	0.81	Ascomycota; Sordariomycetes	Sordariomycetes
WB5151	KY940517	*Virgaria nigra* NBRC 9453 (AB670716)^83^	99	Rhizome	0.41	Ascomycota; mitosporic Ascomycota	*Virgaria* sp.
WB5152	KY940518	*Volutella consors* CBS 139.79 (KM231768)^84^	98	Rhizome	0.81	Ascomycota; Sordariomycetes; Hypocreales	*Volutella* sp.
WB5153	KY940519	*Xenoacremonium falcatus* CBS 400.85 (KM231832)^84^	99	Rhizome	0.41	Ascomycota; Sordariomycetes; Hypocreales	*Xenoacremonium* sp.
WB5150	KY940516	Xylariales W5c8110H (GQ924056)^85^	95	Rhizome	2.44	Ascomycota; Sordariomycetes; Xylariales	Xylariales

In further analyses, 49 representative morphotypes belonged to four classes of the Ascomycota phylum, including Dothideomycetes, Eurotiomycetes, Leotiomycetes, and Sordariomycetes. Most of the isolates (n = 28) from *D. versipellis* belonged to Sordariomycetes class in this study. This class was represented by seven orders: Glomerellales (7 isolates), Hypocreales (13 isolates), Diaporthales (2 isolates), Xylariales (4 isolates), Magnaporthales (1 isolate), Ophiostomales (1 isolate), Sordariales (1 isolate); and 13 genera: *Acremonium*, *Arthrinium*, *Colletotrichum*, *Cylindrocarpon*, *Dactylonectria*, *Diaporthe*, *Fusarium*, *Hypoxylon*, *Ilyonectria*, *Pestalotiopsis*, *Pestalotiopsis*, *Volutella* and *Xenocremonium*. Six isolates (WB5119, WB5133, WB5147, WB5148, WB5149 and WB5150) had no sequence similarities with any reference species from the GenBank database.

Ten isolates were assigned to Dothideomycetes class, comprising three orders: Pleosporales (6 isolates), Capnodiales (3 isolates) and Venturiales (1 isolate) and eight genera (*Alternaria*, *Cladosporium*, *Ochroconis*, *Microsphaeropsis*, *Phoma*, *Pseudocercospora*, *Pyrenochaeta* and *Ramichloridium*). Three isolates were assigned to Eurotiomycetes class and Chaetothyriales order, representing the genera *Exophiala*, *Minimelanolocus* and *Phialophora*. Finally, two isolates were assigned to Letiomycetes class. One (WB5144) was classified as *Rhexocercosporidium* genus of the Helotiales order. No sequence similarity with any reference species was detected in GenBank for the WB5128 isolate.

The present data show that *D. versipellis* roots and rhizomes contain a rich diversity of endophytic fungi, and we found that the most ubiquitous phylum of fungi is Ascomycota, which is reportedly among the most prevalent group of eukaryotes globally^24,25^. In addition, Sordariomycetes was the most prevalent class of endophytic species in the present study, followed by Dothideomycetes, Eurotiomycetes, and Leotiomycetes, as shown previously. We also found that 77.4% of endophytic fungi are present in roots and rhizomes of *D. versipellis*, and only 22.6% of fungal isolates were found in stems and leaves. *Colletotrichum* is a common fungal genus^26^ and was abundant in the stems and leaves, but was absent in roots and rhizomes. *Cylindrocarpon*, *Fusarium*, *Ilyonectria*, and *Rhizoctonia* only colonized roots and rhizomes, whereas *Alternaria*, *Arthrinium*, *Mucor*, *Pestalotiopsis*, *Phialophora*, *Phoma*, *Rhizoctonia* were exclusively detected in roots. Another 19 isolates only colonized rhizomes, and *Acremonium* and *Ochroconis* were exclusively present in leaves. *Pseudocercospora*, *Ramichloridium* only colonized stems. Based on these varying spatial distributions of endophyte communities in *D. versipellis*, we suggested that these microbiotas have adapted to distinct tissue microenvironments, resulting in clear tissue specificity among endophytic fungi in *D. versipellis*, as indicated in a previous study of Indian medicinal plants^27,28^.

Additionally, the isolates WB5143 (*Ramichloridium* sp., Fig. 1c), WB5104 (Ascomycota) and WB5136 (*Cadophora* sp.) have darkly pigmented and septate hyphae of thick walls. These are referred to as dark septate fungi (DSE) and were isolated from roots. Jumpponen & Trappe suggested that DSE frequently colonize roots of mycorrhizal or nonmycorrhizal plants and play unique roles in terrestrial ecosystems^29^. However, in contrast with the common root tissue habitat of DSE, *Ramichloridium* sp. (WB5143) was isolated from stems of plants.

### Antimicrobial activity of ethanolic fraction of culture supernatants of endophytic fungal species

In this study, antimicrobial-producing fungi belonged to the genera *Fusarium, Cladosporium, Ilyonectria, Microsphaeropsis, Cadophora, Phoma, Rhizoctonia, Virgaria*. In addition, the ethanolic extracts of two unidentified isolates also inhibited the microbial growth (Table 3).

**Table 3:** Antibacterial and antifungal activities of endophytic fungi from *D. versipellis* against five pathogens.

Isolate No	Taxa (accession number)	Inhibition zone in diameter on Petri plates (mm)				
		*S. aureus*	*E. coli*	*B. subtilis*	*A. fumigatus*	*C. tropicalis*
WB5106	*Cladosporium* sp. (KY940474)	10.9 ± 0.3	10.8 ± 0.5	11.0 ± 0.3	—	19.1 ± 0.7
WB5121	*Fusarium* sp. (KY940489)	18.7 ± 0.9	21.3 ± 0.7	10.0 ± 0.1	7.3 ± 0.3	—
WB5127	*Ilyonectria* sp. (KY940494)	—	—	7.5 ± 0.4	—	21.0 ± 0.3
WB5134	*Microsphaeropsis* sp. (KY940501)	7.3 ± 0.5	9.7 ± 0.2	8.0 ± 0.5	—	—
WB5136	*Cadophora* sp. (KY940503)	15.0 ± 0.4	14.0 ± 0.3	—	—	8.0 ± 0.5
WB5138	*Phoma* sp. (KY940506)	10.2 ± 0.5	10.3 ± 0.2	15.5 ± 0.3	—	—
WB5145	*Rhizoctonia* sp. (KY940512)	10.9 ± 0.2	17.8 ± 0.2	—	—	—
WB5147	Sordariales (KY940514)	9.6 ± 0.3	10.8 ± 0.4	—	—	13.7 ± 0.2
WB5148	Sordariomycetes (KY940515)	25.0 ± 0.5	—	10.0 ± 0.4	7.0 ± 0.5	18.0 ± 0.3
WB5151	*Virgaria* sp. (KY940517)	9.6 ± 0.3	—	—	—	—
Positive control-1	Ampicillin	17.0 ± 0.3	18.6 ± 0.2	21.5 ± 0.3	—	—
Positive control-2	Fluconazole	—	—	—	25.0 ± 0.3	18.1 ± 0.2
Negative control	10% DMSO	—	—	—	—	—

Endophytic strains of *Fusarium* are well-known producers of various metabolites screened in the host plants^30^; the commercially important drug precursor PTOX was originally found in the endangered genus *Dysosma*^21^ but is also produced by the endophytic *F. oxysporum* from *Juniperus recurva* plants^23^. Other natural agents include Taxol which was originally found in *Taxus* plants and was produced by endophytic *F. proliferatum* from *Taxus x media*^31^. Additionally, 2-methylbutyraldehyde-substituted α-pyrone, beauvericin, and subglutinol A and B are dominant antimicrobial compounds that are produced by endophytic *Fusarium* spp. isolated from medicinal plants^32–34^. Most members of the genus *Cladosporium* also produce antimicrobial compounds, and *C. uredinicola* from *Tinospora cordifolia* was found to possess anti-insect properties, potentially protecting plants against insect pests^35^. In the present study, *Cladosporium* sp. (WB5106) exhibited high antimicrobial activity against *S. aureus*, *E. coli*, *B. subtilis*, and *C. tropicalis*, but did not show any activity against *A. fumigatus*.

Interestingly, all of the present endophytic fungal strains that produce antimicrobial compounds were isolated from roots or rhizomes of *D. versipellis*. Similar studies had also showed medicinal plants with antifungal, antibacterial, anticancer, and antioxidant activities may provide more feasible opportunities to isolate and culture endophytic fungal producers^6,36^. However, further studies are required to characterize dynamic changes of endophytic communities^6^ and uncultured fungi^30^ and to confirm fungal tissue specificity in *D. versipellis*.

### Screening of PTOX-producing fungi

**Figure 2:**
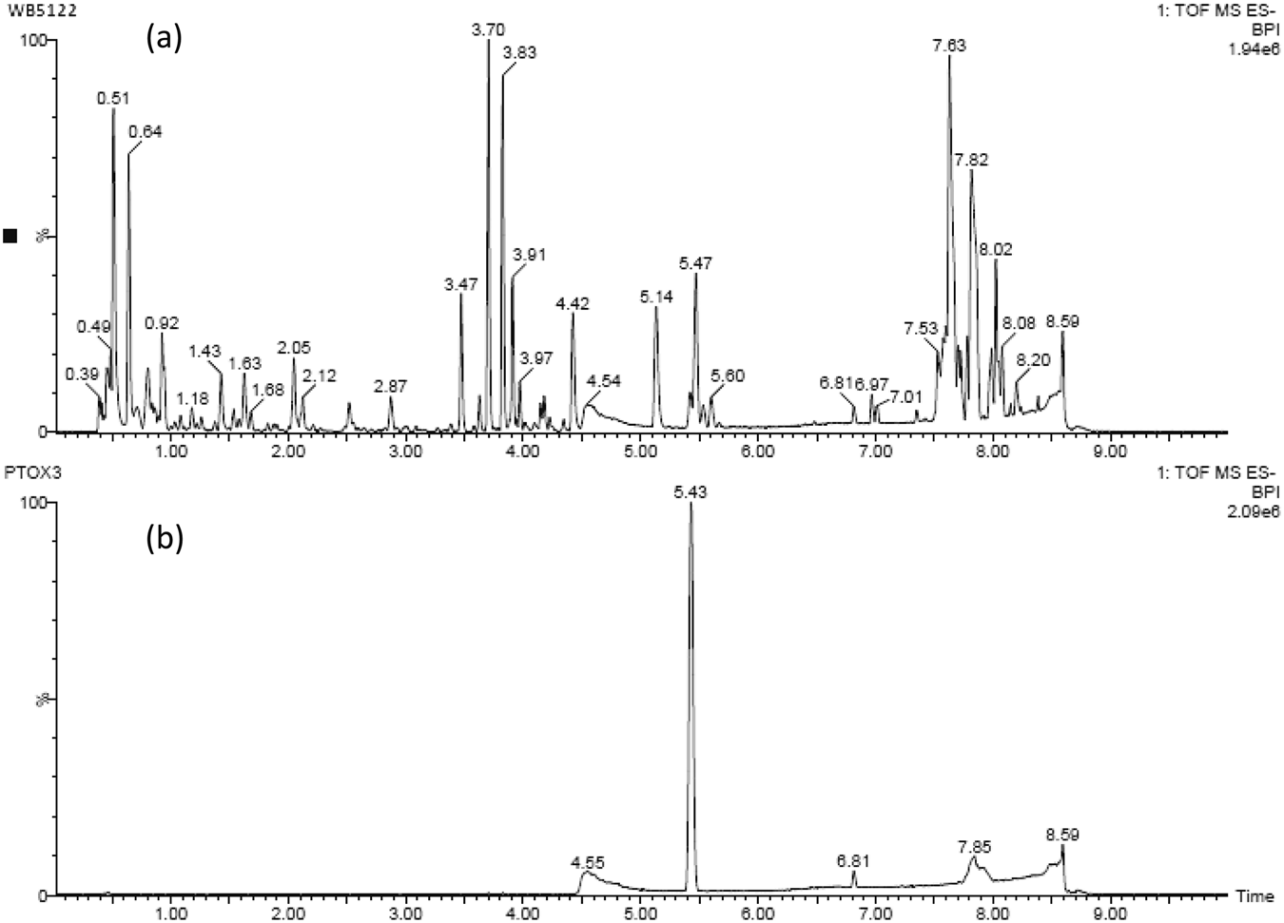
Representative base peak ion chromatograms of *Fusarium* sp. (WB5122) extract (**a**) and standard podophyllotoxin (PTOX) samples (**b**) from UHPLC-QTOF-MS/MS analyses performed in negative ionmode.

**Figure 3:**
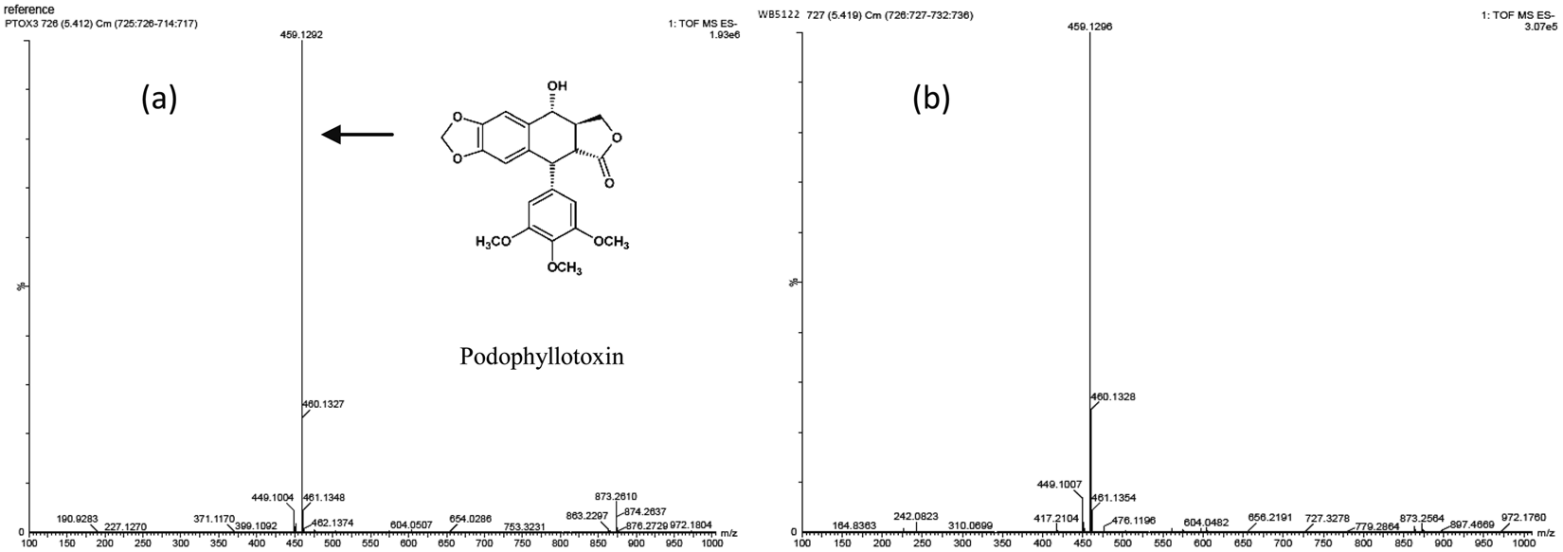
MS spectra of PTOX; standard podophyllotoxin (**a**); fungal PTOX isolated from *Fusarium* sp. WB5122 (**b**); the *arrow* indicates the molecular ion of PTOX at *m*/*z* 459.12 (MH^+^).

Crude extracts of endophytic fungi were screened for fungal PTOX using HPLC and UPLC–QTOF MS analyses. In these analyses, PTOX from *Fusarium* sp. WB5121 and WB5122 had retention times that corresponded with the standard PTOX (Fig. 2) and corresponding yields were 277 and 1.25 µg/g (wet weight of crude extracts), respectively, after culture in 200 mL of potato dextrose broth (PDB) at 26 °C ± 2 °C with shaking at 125 rpm for 10 days. Associated MS spectra showed the same peak MH^+^ at *m*/*z* 459.12 for standard and fungal PTOX from *Fusarium* sp. WB5122, and that of the fungal PTOX from *Fusarium* sp. WB5121 yielded a peak MH^+^ at *m*/*z* 459.13 (Fig. 3), indicating the presence of endogenous PTOX in isolates of *Fusarium* sp. WB5122 and WB5121 strains.

In conclusion, *D. versipellis* harbors a rich and diverse range of endophytic fungi and provides a fungal resource for the study of PTOX and other unique secondary metabolites. Among the present endophytic fungi, 18.9% and 3.7% of strains produced antimicrobial and anticancer metabolites, respectively. Hence, future studies of metabolic pathways, mutual relationships, and fungal species identification are warranted.

## Materials and Methods

### Collection of plant material

The wild plant samples of *D. versipellis* were collected from Yongfu county, Guangxi province of China (109°36′E; 24°37′N). Samples were placed in polyethylene bags, labeled, transported to the laboratory, and refrigerated at 4 °C, as described previously^37^. Plant specimens were identified by Dr. Tan and were preserved in the herbarium of the Guangxi Botanical Garden of Medicinal Plants.

### Fungal isolation and cultivation

Endophytic fungi were isolated from stems, leaves, and roots of plants. Procedures for surface sterilization of plant tissues and isolation and cultivation of fungi are described by Tan *et al*.^38^. Briefly, stems, leaves, and roots were separated from plants, were washed thoroughly in running tap water, and were surface-sterilized in a sequence of 70% ethanol (*v*/*v*) for 30 s and sodium hypochlorite solution (2.5%, *v*/*v*) for 5 min. All tissues were then rinsed three times with sterile distilled water and were surface-dried with sterile filter paper. Subsequently, 0.5 × 0.5-cm pieces were excised using a sterile blade and were placed on PDA containing 50-µg/mL oxytetracycline and 50-µg/mL streptomycin. Nine segments were plated per Petri dish (90-mm diameter). Petri dishes were then wrapped in parafilm and were incubated at 25 °C in the dark for more than one week. Samples were checked daily and colonies were routinely isolated, purified, and maintained in PDA for identification and antimicrobial assays. Pure endophytic fungi were finally photographed and preserved in the laboratory of Mycology, Guangxi Botanical Garden of Medicinal Plants.

### DNA extraction, PCR amplification, sequencing, and molecular identification

To produce fungal mycelia, all strains were grown on PDA plates at 25 °C for 10 days. Mycelia were scraped using sterile pipette tips and were then freeze-dried, and DNA from endophytic fungi were then extracted using E.Z.N.A.TM Fungal DNA Mini Kits (Omega Bio-tek, Norcross, USA) according to the manufacturers’ instructions for use as templates in polymerase chain reactions (PCR). The primers ITS1 (5′-TCCGTAGGTGAACCTGCGG-3′) and ITS4 (5′-TCCTCCGCTTATTGATATGC-3′) were constructed for molecular phylogenetic studies and were used to amplify ribosomal internal transcribed spacers (ITS)^39^. The PCR mixture (50 µL) contained 25 µL of Taq PCR Master Mix (Qiagen, Bejing), 2 µL of each primer at 5 µM, 19 µL of H_2_O, and 2 µL of genomic DNA. PCR were performed using a thermal cycler (BioRAD) with an initial denaturation step at 95 °C for 3 min, followed by 35 cycles of 94 °C for 1 min, 55 °C for 30 s, 72 °C for 1 min, and then a final extension step at 72 °C for 7 min. Subsequently, 5-µL PCR products were analyzed electrophoretically in 1% (*w*/*v*) agarose gels stained with ethidium bromide. After visual inspection under UV light, 45-µL aliquots of PCR products were purified and sequenced at the Shanghai Sangon Biological Engineering Technology & Services Co. Ltd. Sequences were then compared with ITS sequences from reliable isolates listed in the NCBI database (http://www.ncbi.nlm.nih.gov). Only sequence matches with high similarity to those published in previous studies were included in analyses. All identified isolates were categorized at genus or family levels according to the ownership criterion as follows: species of the same genera have sequence similarity (SS) of >95% and those of the same families had SS of <95%^6,40^. The sequences obtained in this study were previously submitted to the GenBank database with accession numbers from KY940469 to KY940519.

### Crude extract preparation of fungal fermentation broth

Fifty-three strains were precultured on PDA (potato extract, 200 g/L; dextrose, 20 g/L) for 7 days, and five plugs (6 mm of diameter) of each fungus were then pre-inoculated into 500-mL Erlenmeyer flask containing 200-mL PDB containing 200 g/L potato extract and 20 g/L dextrose. All cultures were incubated on a rotary shaker (125 rpm) at 26 °C ± 2 °C in the dark for 10 days. Cultures were then filtered to collect fermentation broth and wet mycelia were discarded. Fermentation broth was extracted with four volumes of ethanol for one day and filtrates were further concentrated *in vacuo* to remove organic solvent^41^. Concentrates were then volatilized in a water bath at 60 °C and dried residues and were finally stored at −20 °C. Crude extracts were diluted with 10% dimethyl sulfoxide (DMSO) to 10 mg/mL and were sterilized by filtration using a Millipore filter (0.22 µm) prior to antimicrobial assays.

### Antimicrobial activity

Five pathogens, including the fungi *A. fumigatus* and *C. albicans* and bacteria *E. coli*, *B. subtilis*, and *S. aureus*, were used to test antimicrobial activities of 53 crude fungal EtOH extracts, and inhibitory effects were assayed using the agar diffusion method with 10-mg/mL extracts at 100 µg/disk. Ampicillin sodium (100 µg/disk) and fluconazole (25 µg/disk) were used as positive antimicrobial controls and 10% DMSO was used as a negative control. Antimicrobial activities were determined according to diameters of inhibition zones (ZI) and experiments were repeated three times.

### Determination of PTOX-producing fungi

PTOX-producing endophytic fungi were screened using HPLC^22,23^ analyses and the agent was identified using UPLC–QTOF MS. In these experiments, crude extracts of fungal isolates were dissolved in 1 mL of 80% methanol (*v*/*v*) and were filtered through 0.22-µm syringe filters prior to HPLC analyses (Agilent 1260, USA), which were performed using a Zorbax SB-C_18_ column (5 µm, 4.6 mm × 250 mm; Agilent, USA). Gradient elution was then performed with acetonitrile/H_2_O binary solvent-delivery gradient elution at a flow rate of 1.0 mL/min as follows: 0–20 min, 20% acetonitrile; 20–25 min, 60% acetonitrile; 25–30 min, acetonitrile; volume fraction. Analytes were detected at 207 nm and injection volumes for all fungal methanol extracts and PTOX standard were 20 and 5 µL, respectively. PTOX standard was purchased from Sigma-Aldrich Corporation (St. Louis, Missouri, USA).

Fungal PTOX was further identified using a UPLC–QTOF MS system (Waters, USA) as described previously^42^. Briefly, chromatographic separation was performed with an Acquity UPLC HSS T3 C_18_ column (1.8 μm, 2.1 mm × 100 mm) with an injection volume of 0.3 µL and a binary gradient elution mixture comprising water with 0.1% formic acid (A) and 0.1% formic acid in acetonitrile (B) as follows: 0–3.5 min, 10–35% B; 3.5–5.5 min, 35–40% B; 5.5–6.5 min, 40–60% B; 6.5–8.0 min, 60–90% B; 8.1–10 min, 10% B. The mobile phase was applied at a flow rate of 0.5 mL/min and the temperature of the column oven was set to 35 °C.

The MS was operated in negative ion mode and was set to total ion chromatogram mode with the following mass conditions: capillary voltage = 2500 V, cone voltage = 40 V, low collision energy = 6 V, source temperature = 100 °C, desolvation temperature = 400 °C, and desolvation gas flow = 800 L/h. Data acquisition and processing were conducted using MassLynx version 4.1 (Waters, Manchester, UK).

### Statistical analyses

Colonization rates (CR%) of fungal strains isolated from *D. versipellis* were calculated as follows: CR% = (*Nsc*/*Nss*) × 100, where *Nsc* represents the number of segments infected by fungi and the *Nss* represents the total number of segments investigated^43^. Isolation rates (IR%) of the strains were calculated as follows: IR% = (*Ni*/*Nt*) × 100, where Ni represents the number of segments from which fungal species were isolated and Nt is the total number of segments incubated^44^. The diversity of fungal species from *D. versipellis* was evaluated using the Shannon–Weiner Index (*H′*) with the following formulas:

$$H^{\prime} =-{\rm{\Sigma }}(Pi\times {\rm{l}}{\rm{n}}\,Pi)\,(Pi=ni/N),$$

where *ni* represents the numbers of individuals and *N* represents the total number of individuals^45^. All statistical analyses were performed using SPSS 19.0 (SPSS Inc., Chicago, IL, USA).

## Electronic supplementary material

Supplementary information
