# Author Correction: Diversity and bioactive potential of culturable fungal endophytes *of Dysosma versipellis*; a rare medicinal plant endemic to China

**DOI:** 10.1038/s41598-018-31009-0

**Published:** 2018-08-20

**Authors:** Xiao-ming Tan, Ya-qin Zhou, Xiao-lei Zhou, Xiang-hua Xia, Ying Wei, Li-li He, Hong-zhen Tang, Li-ying Yu

**Affiliations:** 1 Guangxi University of Chinese Medicine, Nanning, 530200 China; 2 Guangxi Botanical Garden of Medicinal Plant, Nanning, 530023 China

Correction to: *Scientific Reports* 10.1038/s41598-018-24313-2, published online 12 April 2018

The original version of this Article contained errors in the spelling of the authors Xiao-ming Tan, Ya-qin Zhou, Xiao-lei Zhou, Xiang-hua Xia, Li-li He, Hong-zhen Tang and Li-ying Yu which were incorrectly given as Xiaoming Tan, Yaqin Zhou, Xiaolei Zhou, Xianghua Xia, Lili He, Hongzhen Tang and Liyin Yu respectively.

These errors have now been corrected in the PDF and HTML versions of the Article, and in the accompanying Supplementary Information file.

