# Supplementary material for: Diversity and bioactive potential of culturable fungal endophytes of Dysosma versipellis; a rare medicinal plant endemic to China
Source: Sci Rep. 2018 Apr 12;8:5929. doi: 10.1038/s41598-018-24313-2 (PMC5897559; doi:10.1038/s41598-018-24313-2)
Supplement: Supplementary file 1 — Supplementary information [file 41598_2018_24313_MOESM1_ESM.docx]

**Supplementary Information**

**Diversity and bioactive potential of culturable fungal endophytes of** ***Dysosma versipellis*; a rare medicinal plant endemic to China**

Xiao-ming Tan^1✉^, Ya-qin Zhou^2^, Xiao-lei Zhou^2^, Xiang-hua Xia^2^, Ying Wei^2^, Li-li He^2^, Hong-zhen Tang^1✉^ & Li-ying Yu^2^

^1^Guangxi University of Chinese Medicine, Nanning, 530200, China

^2^Guangxi Botanical Garden of Medicinal Plant, Nanning, 530023, China.

^✉^ Correspondence to [txm1978@126.com](mailto:txm1978@126.com) or hzhent2012@126.com.

**TABLE OF CONTENTS**

**Supplemental Figure 1. Habitat of *D. versipellis* and its endophytic fungi.**

**Supplemental Figure 2.** **Representative base peak ion chromatograms of *Fusarium* sp. (WB5122) extract (a) and standard podophyllotoxin (PTOX) samples (b) from UHPLC-QTOF-MS/MS analyses performed in negative ionmode.**

**Supplementary Figure 3. MS spectra of PTOX; standard podophyllotoxin (a); fungal PTOX isolated from *Fusarium* sp. WB5122 (b); the *arrow* indicates the molecular ion of PTOX at *m*/*z* 459.12 (MH^+^).**

**Supplemental Table 1. Endophytic isolates from *D. versipellis* tissues.**

**Supplemental Table 2. Culturable endophytic fungi from *D. versipellis* and corresponding isolation rates (IR%).**

**Supplemental Table 3.** **Antibacterial and antifungal activities of endophytic fungi from *D. versipellis* against five pathogens.**



**Supplemental Figure 1 Habitat of *D. versipellis* and its endophytic fungi.** Adult plants of *D. versipellis* (Bar = 20 mm; a) growing among hillside shrubs (b) and representative fungal morphotypes isolated from *D. versipellis* growing on potato dextrose agar (PDA) for 2 weeks at 26°C (c)



**Supplemental Figure 2.** **Representative base peak ion chromatograms of *Fusarium* sp. (WB5122) extract (a) and standard podophyllotoxin (PTOX) samples (b) from UHPLC-QTOF-MS/MS analyses performed in negative ionmode.**



**Supplementary Figure 3. MS spectra of PTOX; standard podophyllotoxin (a); fungal PTOX isolated from *Fusarium* sp. WB5122 (b); the *arrow* indicates the molecular ion of PTOX at *m*/*z* 459.12 (MH^+^)**

**Supplementary Table 1 Endophytic isolates from *D. versipellis* tissues**

| Tissues | Segments examined | Segments  infected | Total isolates | Endophytic species | Total CR% | Total  IR% | Shannon_*H′* |
| --- | --- | --- | --- | --- | --- | --- | --- |
| Root | 190 | 58 | 62 | 19 | 30.50% | 32.60% | 2.433 |
| Rhizome | 246 | 97 | 104 | 22 | 39.40% | 42.30% | 2.728 |
| Stem | 45 | 31 | 33 | 6 | 68.80% | 73.30% | 1.330 |
| Leaf | 63 | 23 | 25 | 6 | 36.50% | 39.70% | 1.242 |
| Total | 544 | 209 | 224 | 53 |  |  |  |

**Supplementary Table 2 Culturable endophytic fungi from *D. versipellis* and corresponding isolation rates (IR%)**.

| Fungal isolate | Accession number | Closest relatives in NCBI | ITS identity (%) | Tissue | IR % | Phylum; Class; Order | Classification |
| --- | --- | --- | --- | --- | --- | --- | --- |
| WB5101 | KY940469 | *Acremonium nepalense* CBS 113254 (DQ825972) ^24^ | 99 | Leaf | 1.12 | Ascomycota; Sordariomycetes; Glomerellales | *Acremonium* sp. |
| WB5102 | KY940470 | *Alternaria alternata* CBS 112018 (AY673074) ^25^ | 99 | Root | 0.53 | Ascomycota; Dothideomycetes; Pleosporales | *Alternaria* sp. |
| WB5103 | KY940471 | *Arthrinium arundinis* CBS 114316 (KF144884) ^26^ | 99 | Root | 0.53 | Ascomycota; Sordariomycetes; Xylariales | *Arthrinium* sp. |
| WB5104 | KY940472 | Ascomycota P7 (AY265338) ^27^ | 80 | Rhizome | 0.41 | Ascomycota | Ascomycota |
| WB5105 | KY940473 | Ascomycota (JX427054) ^28^ | 84 | Root | 0.53 | Ascomycota | Ascomycota |
| WB5106 | KY940474 | *Cladosporium uredinicola* SACCR 040661 (AY251071) ^29^ | 99 | Rhizome | 0.41 | Ascomycota; Dothideomycetes; Capnodiales | *Cladosporium* sp. |
| WB5107 | KY940475 | *Colletotrichum excelsum-altitudum* CGMCC 3.15131 (JX625182) ^30^ | 99 | Leaf | 1.12 | Ascomycota; Sordariomycetes; Glomerellales | *Colletotrichum* sp. |
| WB5108 | KY940476 | *Colletotrichum gigasporum* P1982 (KT269249) ^31^ | 99 | Stem | 2.22 |  | *Colletotrichum* sp. |
| WB5109 | KY940477 | *Colletotrichum gloeosporioides* CBS 119204 (JX010150) ^32^ | 99 | Leaf | 19.04 |  | *Colletotrichum* sp. |
| WB5110 | KY940478 | *Colletotrichum karstii* CGMCC 3.15123 (JX625163) ^30^ | 99 | Leaf | 1.12 |  | *Colletotrichum* sp. |
| WB5111 | KY940479 | *Colletotrichum siamense* GM29 (KC512127) ^33^ | 100 | Stem | 2.22 |  | *Colletotrichum* sp. |
| WB5113 | KY940481 | *Cylindrocarpon liriodendra* CBS 117640 (DQ178166) ^34^ | 99 | Rhizome | 0.41 | Ascomycota; Sordariomycetes; Hypocreales | *Cylindrocarpon* sp. |
| WB5114 | KY940482 | *Cylindrocarpon pauciseptatum* Cy196 (JF735305) ^35^ | 99 | Root | 0.53 |  | *Cylindrocarpon* sp. |
| WB5131 | KY940498 | *Cylindrocarpon* sp. YIMPH30026 (KP230827) ^36^ | 97 | Root | 0.53 |  | *Cylindrocarpon* sp. |
| WB5115 | KY940483 | *Dactylonectria alcacerensis* CBS 129087 (NR_121498) ^35^ | 99 | Rhizome | 0.41 | Ascomycota; Sordariomycetes; Hypocreales | *Dactylonectria* sp. |
| WB5149 | KY940504 | *Uncultured Diaporthales* R77p1 (GU327455) ^37^ | 94 | Root | 0.53 | Ascomycota; Sordariomycetes; Diaporthales | Diaporthales |
| WB5116 | KY940484 | *Diaporthe perjuncta* CBS 109745 (KC343172) ^38^ | 96 | Rhizome | 0.41 | Ascomycota; Sordariomycetes;  Diaporthales | *Diaporthe* sp. |
| WB5117 | KY940485 | *Diaporthe* sp. HKB37 (DQ092525) ^39^ | 96 | Rhizome | 0.41 |  | *Diaporthe* sp. |
| WB5118 | KY940486 | *Exophiala* sp. AS29-1 (AB752282) ^40^ | 99 | Rhizome | 3.65 | Ascomycota; Eurotiomycetes; Chaetothyriales | *Exophiala* sp. |
| WB5120 | KY940488 | *Fusarium nematophilum* BBA 70838 (HQ897786) ^41^ | 99 | Rhizome | 2.43 | Ascomycota; Sordariomycetes; Hypocreales | *Fusarium* sp. |
| WB5121 | KY940489 | *Fusarium oxysporum* ERP-10 (JN222394) ^42^ | 99 | Root | 0.53 |  | *Fusarium* sp. |
| WB5122 | KY940468 | *Fusarium solani* ATCC 56480 (FJ345352) ^43^ | 100 | Root | 0.53 |  | *Fusarium* sp. |
| WB5123 | KY940490 | *Hypoxylon fragiforme* 22 (JN198512) ^44^ | 99 | Rhizome | 0.41 | Ascomycota; Sordariomycetes; Xylariales | *Hypoxylon* sp. |
| WB5124 | KY940491 | *Ilyonectria coprosmae* CBS 119606 (JF735260) ^35^ | 96 | Root | 10.53 | Ascomycota; Sordariomycetes; Hypocreales | *Ilyonectria* sp. |
| WB5125 | KY940492 | *Ilyonectria macrodidyma* K6 (JF807395) ^45^ | 99 | Rhizome | 1.62 |  | *Ilyonectria* sp. |
| WB5126 | KY940493 | *Ilyonectria robusta* CBS 117815 (JF735266) ^35^ | 96 | Rhizome | 5.69 |  | *Ilyonectria* sp. |
| WB5127 | KY940494 | *Ilyonectria torresensis* CBS 112598 (JF735351) ^35^ | 99 | Rhizome | 1.62 |  | *Ilyonectria* sp. |
| WB5128 | KY940495 | Leotiomycetes AK1466 (JQ759764) ^46^ | 89 | Root | 0.53 | Ascomycota; Leotiomycetes | Leotiomycetes |
| WB5129 | KY940496 | *Minimelanolocus aquaticus* 15-0414 (KR215607) ^47^ | 97 | Rhizome | 2.03 | Ascomycota; Eurotiomycetes; Chaetothyriales | *Minimelanolocus* sp. |
| WB5130 | KY940497 | *Mucor* sp. CY118 (HQ607969) ^48^ | 95 | Root | 0.53 | Zygomycota; Zygomycetes;  Mucorales | *Mucor* sp. |
| WB5132 | KY940499 | *Ochroconis* cf. constricta CBS 124172 (GQ426969) ^49^ | 99 | Leaf | 1.12 | Ascomycota; Dothideomycetes; Venturiales | *Ochroconis* sp. |
| WB5133 | KY940500 | *Ophioceras* sp. F2224 (KU747946) ^50^ | 94 | Stem | 2.22 | Ascomycota; Sordariomycetes; Magnaporthales | Magnaporthales |
| WB5119 | KY940487 | OphiostomatalesF1732 (KU747803) ^50^ | 97 | Stem | 2.22 | Ascomycota; Sordariomycetes; Ophiostomatales | Ophiostomatales |
| WB5134 | KY940501 | *Microsphaeropsis* sp. S4A1ACS (KY305064)^51^ | 99 | Root | 0.53 | Ascomycota; Dothideomycetes; Pleosporales | *Microsphaeropsis* sp. |
| WB5135 | KY940502 | *Pestalotiopsis oryzae* CBS 111522 (KM199294) ^52^ | 99 | Root | 0.53 | Ascomycota; Sordariomycetes; Xylariales | *Pestalotiopsis* sp. |
| WB5136 | KY940503 | *Phialophora mustea* BAN-C4 (JN123359) ^53^ | 99 | Root | 0.53 | Ascomycota; Eurotiomycetes; Chaetothyriales | *Phialophora* sp. |
| WB5137 | KY940505 | *Phoma putaminum* CBS 372.91 (GU237843) ^54^ | 99 | Root | 0.53 | Ascomycota; Dothideomycetes; Pleosporales | *Phoma* sp. |
| WB5138 | KY940506 | *Phoma selaginellicola* CBS 122.93 (GU237762) ^54^ | 99 | Root | 0.53 |  | *Phoma* sp. |
| WB5139 | KY940507 | *Phyllosticta* sp. MUCC0547 (AB454364) ^55^ | 99 | Rhizome | 0.41 | Basidiomycota; Agaricomycetes; Agaricales | *Phyllosticta* sp. |
| WB5140 | KY940508 | *Psathyrella candolleana* P73 (AM712281) ^56^ | 99 | Rhizome | 0.41 | Basidiomycota; Agaricomycetes; Agaricales | *Psathyrella* sp. |
| WB5141 | KY940509 | *Pseudocercospora humuli* CPC 11358 (GU214676) ^57^ | 99 | Stem | 26.7 | Ascomycota; Dothideomycetes; Capnodiales | *Pseudocercospora* sp. |
| WB5112 | KY940480 | *Pyrenochaeta* sp. P2916 (KT270113) ^31^ | 98 | Root | 1.05 | Ascomycota; Dothideomycetes; Pleosporales | *Pyrenochaeta* sp. |
| WB5142 | KY940510 | *Pyrenochaeta* sp. CBS 135108 (KF251149) ^58^ | 97 | Leaf | 1.12 | Ascomycota; Dothideomycetes; Pleosporales | *Pyrenochaeta* sp. |
| WB5143 | KY940467 | *Ramichloridium* sp. NC1_3.3F1a (FJ425199) ^59^ | 96 | Stem | 2.22 | Ascomycota; Dothideomycetes; Capnodiales | *Ramichloridium* sp. |
| WB5144 | KY940511 | *Rhexocercosporidium* sp. Dzf14 (EU543257) ^60^ | 99 | Rhizome | 0.41 | Ascomycota; Leotiomycetes;  Helotiales | *Rhexocercosporidium* sp. |
| WB5145 | KY940512 | *Rhizoctonia* sp. Rh183 (JF519833) ^61^ | 99 | Rhizome | 0.81 | Basidiomycota; Agaricomycotina incertae sedis | *Rhizoctonia* sp. |
| WB5146 | KY940513 | *Rhizoctonia* sp. R14 (AY927321) ^62^ | 95 | Root | 0.53 |  | *Rhizoctonia* sp. |
| WB5147 | KY940514 | SordarialesREF169 (JN859389) ^63^ | 95 | Root | 2.11 | Ascomycota; Sordariomycetes; Sordariales | Sordariales |
| WB5148 | KY940515 | Sordariomycetes AK0924 (JQ759304) ^46^ | 88 | Rhizome | 0.81 | Ascomycota; Sordariomycetes | Sordariomycetes |
| WB5151 | KY940517 | *Virgaria nigra* NBRC 9453 (AB670716) ^64^ | 99 | Rhizome | 0.41 | Ascomycota; mitosporic Ascomycota | *Virgaria* sp. |
| WB5152 | KY940518 | *Volutella consors* CBS 139.79 (KM231768) ^65^ | 98 | Rhizome | 0.81 | Ascomycota; Sordariomycetes; Hypocreales | *Volutella* sp. |
| WB5153 | KY940519 | *Xenoacremonium falcatus* CBS 400.85 (KM231832) ^65^ | 99 | Rhizome | 0.41 | Ascomycota; Sordariomycetes; Hypocreales | *Xenoacremonium* sp. |
| WB5150 | KY940516 | Xylariales W5c8110H (GQ924056) ^66^ | 95 | Rhizome | 2.44 | Ascomycota; Sordariomycetes; Xylariales | Xylariales |

**Supplementary Table 3 Antibacterial and antifungal activities of endophytic fungi from *D. versipellis* against five pathogens**

| Isolate No. | Taxa (accession number) | Inhibition zone in diameter on Petri plates (mm) | | | | | | | |
| --- | --- | --- | --- | --- | --- | --- | --- | --- | --- |
|  |  | *S. aureus* | *E. coli* | | *B. subtilis* | | *A. fumigatus* | | *C. tropicalis* |
| WB5106 | *Cladosporium* sp. (KY940474) | 10.9 ± 0.3 | 10.8 ± 0.5 | 11.0 ± 0.3 | | - | | 19.1 ± 0.7 | |
| WB5121 | *Fusarium* sp. (KY940489) | 18.7 ± 0.9 | 21.3 ± 0.7 | 10.0 ± 0.1 | | 7.3 ± 0.3 | | - | |
| WB5127 | *Ilyonectria* sp. (KY940494) | - | - | 7.5 ± 0.4 | | - | | 21.0 ± 0.3 | |
| WB5134 | *Microsphaeropsis* sp. (KY940501) | 7.3 ± 0.5 | 9.7 ± 0.2 | 8.0 ± 0.5 | | - | | - | |
| WB5136 | *Cadophora* sp. (KY940503) | 15.0 ± 0.4 | 14.0 ± 0.3 | - | | - | | 8.0 ± 0.5 | |
| WB5138 | *Phoma* sp. (KY940506) | 10.2 ± 0.5 | 10.3 ± 0.2 | 15.5 ± 0.3 | | - | | - | |
| WB5145 | *Rhizoctonia* sp. (KY940512) | 10.9 ± 0.2 | 17.8 ± 0.2 | - | | - | | - | |
| WB5147 | Sordariales (KY940514) | 9.6 ± 0.3 | 10.8 ± 0.4 | - | | - | | 13.7 ± 0.2 | |
| WB5148 | Sordariomycetes (KY940515) | 25.0 ± 0.5 | - | 10.0 ± 0.4 | | 7.0 ± 0.5 | | 18.0 ± 0.3 | |
| WB5151 | *Virgaria* sp. (KY940517) | 9.6 ± 0.3 | - | - | | - | | - | |
| Positive control-1 | Ampicillin | 17.0 ± 0.3 | 18.6 ± 0.2 | 21.5±0.3 | | - | | - | |
| Positive control-2 | Fluconazole | - | - | - | | 25.0 ± 0.3 | | 18.1 ± 0.2 | |
| Negative control | 10% DMSO | - | - | - | | - | | - | |
